# Patient activation and health behavior decision-making in stroke patients: a longitudinal mediation mechanism of family function and risk perception

**DOI:** 10.3389/fpubh.2026.1725303

**Published:** 2026-05-08

**Authors:** Haixia Li, Xiaoshan Chen, Xiu Zheng, Zhangying Li

**Affiliations:** Department of Neurology, Guangdong Neuroscience Institute, Guangdong Provincial People’s Hospital, Guangdong Academy of Medical Sciences, Southern Medical University, Guangzhou, Guangdong, China

**Keywords:** activation, chain mediation, cross-lag analysis, family functioning, health behavior decision-making, longitudinal studies, perception of recurrence risk, stroke

## Abstract

**Objective:**

To explore the chain mediating effect of family function and recurrence risk perception between activation and health behavior decision-making in stroke patients within 6 months after stroke.

**Methods:**

A total of 254 stroke patients were investigated using the Family APGAR Index, Risk of Recurrence Perception Scale, Activation Scale, and Stroke Behavior Decision Scale before discharge (T1), 3 months after stroke (T2), and 6 months after stroke (T3). Equivalence testing and cross-lag model analysis were employed to analyze the data.

**Results:**

A total of 232 valid questionnaires were collected. Equivalence test results: All variables met the equivalence requirements, indicating that the measurement tools were inter-temporal comparable in dynamic tracking. Cross-lagged model path analysis: T1 activation could significantly predict T2 family function (*β* = 0.28, *p* < 0.01), T2 recurrence risk perception (*β* = 0.20, *p* < 0.01), and T2 health behavior decision (*β* = 0.23, *p* < 0.01). T1 family function could significantly predict T2 recurrence risk perception (*β* = 0.21, *p* < 0.01) and T2 health behavior decision-making (*β* = 0.29, *p* < 0.01); T2 activation significantly positively predicted T3 recurrence risk perception (*β* = 0.19, *p* < 0.01), T2 family function could significantly positively predict T3 recurrence risk perception (*β* = 0.20, *p* < 0.01), and could significantly positively predict T3 health behavior decision (*β* = 0.26, *p* < 0.01). Recurrence risk perception at T2 significantly positively predicted T3 health behavior decision (*β* = 0.17, *p* < 0.01), and health behavior decision at T2 significantly positively predicted T3 activation (*β* = 0.12, *p* < 0.01). Bootstrap analysis showed that T2 family function (*β* = 0.073, *p* < 0.01) and T2 recurrence risk perception (*β* = 0.034, *p* < 0.01) had significant indirect effects on T1 activation in the prediction of T3 health behavior decision.

**Conclusion:**

Within 6 months after a stroke, there is a mutual predictive relationship between activation and health behavior decision-making in stroke patients. Family function and perceived risk of recurrence have a longitudinal mediating effect on the mechanism of activation’s influence on health behavior decision-making.

## Introduction

The global burden of stroke continues to increase. In 2021, there were 11.9 million new stroke cases worldwide, a 70% increase compared with 1990. Moreover, the number of stroke—related deaths rose to 7.3 million, making stroke the third leading cause of death globally (1, 2). Ischemic stroke accounted for the highest proportion (73.33%) (3). The situation of stroke in China is particularly grave. Approximately 3.94 million new cases occur each year, accounting for one – third of the global total. Among these cases, ischemic stroke accounts for 72% and hemorrhagic stroke accounts for 22% (4). In 2020, there were 17.8 million stroke cases among people over 40 years old in China. Around 2.3 million people die of stroke annually, accounting for 20% of the total deaths of residents and ranking as the leading cause of death (5, 6). The incidence of stroke is getting younger. The lifetime risk of stroke is as high as 39.3% among people aged 40 years and above. Among survivors, 75% have suffered functional disabilities, and 40% have severe disabilities (7).

About 70% of strokes are related to modifiable risk factors such as behavior, metabolism, social psychology, and the environment (8). Behavior change intervention is the most direct and effective way to control the risk factors of stroke and prevent its onset and recurrence (9). The process of health behavior change relies on scientific decision-making. The Transtheoretical Model (TTM) conceptualizes behavior change as a dynamic, non-linear process, emphasizing that individuals progress through a continuum of stages—from “pre-contemplation” to “action”—when making health behavior decisions. Throughout this process, decisional balance and self-efficacy serve as the core mechanisms driving behavioral change. For stroke patients, the shift from ambivalence (contemplation) to actual implementation (action) occurs only when their perceived benefits of adopting a health behavior outweigh the perceived costs, coupled with the confidence to overcome associated barriers (10, 11). The Protection Motivation Theory (PMT) further elucidates how patients make decisions based on risk perception. According to PMT, when confronted with a health threat, individuals undergo threat appraisal (perceived severity and susceptibility) and coping appraisal (self-efficacy and response efficacy) to form protective motivation. In stroke rehabilitation, patients’ perception of recurrence risk (threat appraisal) motivates them to adopt protective behaviors. Meanwhile, support from family functioning—such as emotional backing and information sharing—enhances patients’ self-efficacy (coping appraisal), thereby encouraging more informed health behavior decisions, such as medication adherence and consistent engagement in rehabilitation exercises (12, 13). Indeed, health behavior decision-making is closely linked to patients’ risk perception, as they integrate perceived disease risk, self-efficacy for behavior change, and external environmental factors into their decision-making process (14).

According to the Health Action Process Approach theory, the generation of healthy behaviors decision-making can be divided into three periods: the pre-intention period, the intention period, and the action period (15). On the premise of making corresponding behavioral decisions, activation is an important driving factor that helps patients overcome the obstacles to implementing the action plan and achieving health behavior change.

Studies have shown that the level of family function is closely related to patients’ enthusiasm for participating in rehabilitation (16). As patients’ enthusiasm increases, family function is strengthened through mechanisms such as emotional support, information sharing, and role collaboration, thus forming a virtuous cycle (17). This interaction enhances the patient’s ability to perceive the risk of stroke recurrence. Especially when family function is good, patients are more sensitive to symptom changes and understand the importance of preventive measures.

At the same time, a positive family environment encourages patients to make more scientific health behavior decisions by reducing anxiety and improving self-efficacy, such as taking medicine regularly and adhering to rehabilitation training, so as to reduce the risk of recurrence and improve long-term prognosis (18). Therefore, family function and recurrence risk perception may play a mediating role between activation and health behavior decisions.

However, previous studies in this field are limited to cross-sectional studies and only conduct linear regression analysis to compare the static relationship between variables. This approach makes it difficult to provide information on the complete development trajectory of variables and fails to explore the development mechanism of their mediating effect. The Cross-Lagged Panel Model is a statistical model used to analyze the dynamic causal relationship between variables in longitudinal data. By comparing the interaction between variables at different time points, it is beneficial for determining the causal direction between variables, and it is especially suitable for research problems with unclear causal directions. In view of this, this study aims to explore the cross-lagged effect of the chain mediating effect of family function and recurrence risk perception between activation and health behavior decision-making of stroke patients within 6 months after stroke, so as to provide a theoretical basis for clinical staff to dynamically grasp and improve patients’ health behavior decision-making.

## Subjects and methods of study

### Study subjects

A total of 254 stroke patients admitted to our hospital from January 2025 to March 2025 were selected as the research objects by convenience sampling method. Before the start of the study, a written application was submitted to the Office of Medical Ethics of Guangdong Provincial People’s Hospital, and the clinical investigation was carried out after approval (batch number: KY2025-937-01). Inclusion criteria: meeting the clinical diagnostic criteria (19); The first attack; Age 18 and older; They were clearly aware and had basic communication skills, and all signed informed consent. Exclusion criteria: previous movement disorders; Combined with severe heart failure, malignant tumor and other diseases; Patients whose condition is unstable within a few hours of stroke onset; Patients who died or withdrew for other reasons; The questionnaire was missing for more than one time.

### Sample size calculation

The study was conducted using repeated measures with sample size formula (20):


$$n=\frac{2}{\delta^{2}}[\sigma_{\mu }^{2}+\frac{1+(K-1)p_{c}}{K}{\sigma_{e}}^{2}]{\left(U_{\alpha /2}+U_{\beta }\right)}^{2}$$

According to the pilot study, K =3 ${\sigma_{e}}^{2}$ , the error of repeated measurement ${\sigma_{e}}^{2}$ =133.812, the conditional correlation coefficient $\rho_{c}$ = 0.715, and the variance of individual differences $\sigma_{\mu }^{2}$ =172.252, so n= 171. Considering the high sample dropout rate of longitudinal study, the dropout rate was calculated as 20%, n= 171/0.80 = 214, and 254 patients were enrolled in this study.

### Survey tools

#### Questionnaire for basic information

The questionnaire included demographic data (stroke type, gender, age, marital status, place of residence, education level, monthly income, smoking history, drinking history) and disease data (types of past chronic diseases, comorbidities, etc.) of stroke patients.

#### Family care index

The family APGAR index (Adaptation, Partnership, Growth, Affection and Resolve, APGAR) was developed by Smilkstein (21, 22). The scale contains five items, namely, adaptability, growth, cooperation, affection, and affinity. Each item is evaluated on a scale of 0–2 points, and the total score ranges from 0 to 10 points. The Cronbach’s *α* coefficient of the Chinese version of the family APGAR index was 0.808, and the Kappa index was 0.733, indicating good reliability and validity. The Cronbach’s α coefficients of the Chinese version of the family APGAR index in this study were 0.822, 0.855, and 0.796.

#### Recurrence risk perception scale

The recurrence risk perception scale for stroke patients was compiled by Lin Beilei et al. (23). It consists of 2 parts. This study only used the second part of the scale, which includes 3 dimensions: perception of recurrence severity (7 items), perception of recurrence behavior risk factors (6 items), and perception of recurrence disease risk factors (4 items). Each item is assigned 1–3 points from “disagree” to “agree,” and the total score ranges from 17 to 51. The Cronbach’s *α* coefficient of the scale is 0.850. The higher the total score, the higher the patients’ perceived level of recurrence risk. The Cronbach’s α coefficients of the scale in this study are 0.720, 0.769, and 0.802.

#### Patient activation measure-13

The scale was developed by Habbard et al. (24). In this study, Chen Shi-qiao Chinese version was adopted (25). It has 1 dimension and 13 items. A Likert 5-point scoring method was employed, where 0 represents “not applicable.” Scores from 1 to 4 were assigned for responses ranging from “strongly disagree” to “strongly agree.” The original scale scores range from 0 to 52, and higher scores indicate greater positivity. The Cronbach’s *α* coefficients of the scale in this study were 0.821, 0.782, and 0.810.

#### Behavioral decision-making for stroke

The scale was compiled by Lin Beilei et al. (26). It comprises 30 items across 4 dimensions: motivation for behavior change (10 items), intention for behavior change (9 items), decision factors (5 items), and decision balance (6 items). A Likert 5-point scoring method was employed, with scores from 1 to 5 recorded from “strongly disagree” to “strongly agree.” The total scores range from 30 to 150, where higher scores signify better behavioral decision-making. The Cronbach’s *α* coefficients of the scale in this study were 0.902, 0.824, and 0.860.

### Questionnaire recovery and quality control

The questionnaire survey was conducted after obtaining informed consent from the hospital ethics committee and patients. Basic information was collected upon admission. According to the literature review, the cumulative recurrence rates were 7.7 and 9.5% at 3 and 6 months after stroke, respectively (27). Therefore, the family functioning, recurrence risk perception, activation, and cardiac rehabilitation scales were administered before discharge (T1), 3 months (T2), and 6 months (T3) after stroke. Among these, the T1 questionnaire was completed before the patients were discharged from the hospital, while the T2 – T3 questionnaires were collected through WeChat voice, telephone, and home visits. All questionnaires were anonymized to safeguard patients’ privacy. The investigator would present the content of the questionnaire orally, and the patients would select the answers independently. Data collection and entry were carried out by two specially trained researchers. One was responsible for data collection, and the other independently verified the data to ensure the accuracy of data entry. To enhance patient participation, patients were provided with small incentive gifts for completing all questionnaires.

## Statistical methods

Enumeration data were expressed as cases and percentages (%), and measurement data were expressed as mean ± standard deviation. Pearson correlation analysis was employed to examine the correlation between variables, and the Harman single-factor method was used to test for common method variance. Amos 21.0 software was utilized to analyze the mediating effect model, and the maximum likelihood method of variance was applied to test the fitting validity of the model. The model in the PROCESS program was used to test the chain mediation effect, and the Bootstrap method was used to verify the results. MPlus 8.0 software was used to construct a moderated chain mediation model. The bias-corrected percentile Bootstrap method was used to conduct 5,000 samples for the data fitting test of the model and the moderated effect analysis of family function and recurrence risk perception. MPlus 8.0 software was also used for the measurement equivalence test and cross-lag model analysis. When CFI > 0.900, TLI > 0.900, and RMSEA < 0.06, the cross-lag model fitted well. The test level was set at *α* = 0.05.

## Results

### General demographic data

Twenty-two patients were lost to follow-up between T2 and T3. A total of 232 valid questionnaires were collected in this study, including 133 males (57.33%) and 99 females (42.67%). The average age was (61.99 ± 10.23) years, ranging from 33 to 80 years. The detailed information is shown in Table 1.

**Table 1:** General information of the subjects (*n* = 232).

The project	Categories	*n*	%	Items	Categories	*n*	%
Type of stroke	Ischemic	181	78.02	Place of residence	Rural	52	22.41
	Bleeding	51	21.98		Towns	180	77.59
Age (years)	<45	14	6.03	Monthly income (Yuan)	<3,000	11	4.74
	45 ~ 59	94	40.52		3,000 ~ 6,000	84	36.21
	≥60	124	53.45		6,001~	122	52.59
Gender	Male	133	57.33		>10,000	15	6.47
	Female	99	42.67	Smoking history	Yes	31	13.36
Marital status	Married	5	2.16		No	201	86.64
	Unmarried	216	93.10	Drinking history	Yes	76	32.76
	Divorced/widowed	11	4.74		No	156	67.24
Education	Junior high school and below	76	32.76	Comorbid medical history	Yes	162	69.83
	High school, technical secondary school	75	32.33		No	70	30.17
	Junior college	41	17.67				
	Bachelor’s degree or above	40	17.24				

### Common method bias test

The common method deviation test of the three—time data was conducted using the Harman single-factor test. The variances of the three measurements were 20.33, 23.52, and 26.19% respectively, which were less than the critical value level of 40%. Therefore, there is no serious common method bias problem in this study.

### Scores and correlation analysis of variables at three time points in patients

Pearson correlation analysis was employed to examine the scores of each variable at three time points. The results indicated that there was a significant correlation between the two at all three time points (*p* < 0.05), which satisfied the prerequisite of the cross-lag model. The matrix relationship is presented in Table 2.

**Table 2:** Correlation coefficient matrix of variables at three time points (*r* value, *n* = 232).

Items	M ± SD	①	②	③	④	⑤	⑥	⑦	⑧	⑨	⑩	⑪	⑫
① Family function T1	5.82 ± 2.11	1											
② Recurrence risk perception T1	34.24 ± 5.22	0.417^2)^	1										
③ Activation T1	36.89 ± 4.23	0.391^2)^	0.562^2)^	1									
④ Health behavior decision making T1	93.21 ± 12.05	0.457^2)^	0.493^2)^	0.483^2)^	1								
⑤ Family function T2	5.97 ± 2.23	0.563^2)^	0.304^1)^	0.378^2)^	0.463^2)^	1							
⑥ Recurrence risk perception T2	35.14 ± 4.85	0.396^2)^	0.489^2)^	0.462^2)^	0.357^1)^	0.399^2)^	1						
⑦ Activation T2	36.21 ± 5.35	0.304^1)^	0.447^2)^	0.471^2)^	0.478^2)^	0.35^2)^	0.452^2)^	1					
⑧ Health behavior decision making T2	95.28 ± 12.95	0.357^2)^	0.411^2)^	0.438^2)^	0.458^2)^	0.412^2)^	0.468^2)^	0.494^2)^	1				
⑨ Family function T3	6.04 ± 2.18	0.458^2)^	0.278^1)^	0.505^2)^	0.307^1)^	0.452^2)^	0.456^2)^	0.391^2)^	0.462^2)^	1			
⑩ Recurrence risk perception T3	35.60 ± 4.79	0.284^1)^	0.361^2)^	0.312^2)^	0.382^2)^	0.375^2)^	0.418^2)^	0.412^2)^	0.478^2)^	0.373^2)^	1		
⑪ Activation T3	35.12 ± 5.16	0.268^1)^	0.323^2)^	0.406^2)^	0.393^2)^	0.362^2)^	0.423^2)^	0.452^2)^	0.455^2)^	0.392^2)^	0.411^2)^	1	
⑫ Health behavior decision making T3	98.86 ± 13.24	0.311^1)^	0.359^2)^	0.397^2)^	0.404^2)^	0.397^2)^	0.438^2)^	0.403^2)^	0.418^2)^	0.387^2)^	0.445^2)^	0.423^2)^	1

^1)^Indicates *P* < 0.05, ^2)^Indicates *P* < 0.01.

### Equivalence test

In this study, MPlus 8.3 was used to construct models of morphological equivalence, factor loading equivalence, and intercept equivalence. First, a baseline was established through morphological equivalence verification (Model 1). The observed indicators of the same latent variable in the three measurements were set to be consistent across time points. The measurement errors of the same observed indicator were allowed to be correlated across time points, and the remaining parameters were estimated freely. Based on the establishment of morphological equivalence, a weak equivalence model (Model 2) was constructed by applying the cross-time identity constraint of factor loading. This further restricted the factor loading coefficients of the observed variable and the latent variable to be equal. Finally, a strong equivalence model (Model 3) was established using the intertemporal identity constraint of the intercept, and the intertemporal equivalence limit of the intercept parameter was added. ∆CFI ≤ 0.01 and ∆TLI ≤ 0.01 were used as the equivalence test criteria (28). The results in Table 3 show that all the variables meet the equivalence requirements, indicating that the measurement tools are inter-temporal comparable in dynamic tracking and can be used for subsequent cross-lagged path analysis.

**Table 3:** Equivalence model fitting results of three measurements for each variable.

Variables	Model	χ^2^	df	CFI	TLI	RMSEA (95%CI)	SRMR
Family function	Model 1	790.746	372	0.943	0.930	0.048 ([0.044, 0.054])	0.065
	Model 2	806.761	382	0.942	0.928	0.049 ([0.044, 0.054])	0.066
	Model 3	821.673	394	0.941	0.933	0.048 ([0.043, 0.054])	0.066
Recurrence risk perception	Model 1	1013.914	478	0.963	0.948	0.049 ([0.045, 0.054])	0.077
	Model 2	1041.945	490	0.961	0.967	0.049 ([0.045, 0.054])	0.078
	Model 3	1058.406	504	0.958	0.948	0.048 ([0.044, 0.054])	0.079
Activation	Model 1	147.853	65	0.936	0.926	0.051 ([0.040, 0.064])	0.036
	Model 2	159.023	71	0.930	0.931	0.051 ([0.040, 0.064])	0.039
	Model 3	170.205	77	0.929	0.928	0.050 ([0.040, 0.062])	0.040
Health behavior decision making	Model 1	397.162	154	0.950	0.936	0.058 ([0.051, 0.064])	0.046
	Model 2	410.100	164	0.949	0.939	0.056 ([0.050, 0.065])	0.047
	Model 3	434.664	172	0.946	0.938	0.057 ([0.050, 0.064])	0.049

χ^2^ is chi square, df is degrees of freedom, CFI is comparative fit index, TLI is nonbenchmark fit index, RMSEA is root mean square of progressive residuals, SRMR is normalized root mean square residuals.

### Cross-lag analysis

#### Model establishment and fitting

Before the formal construction of the cross-lag model with three time points, four cross-lag models need to be established and tested to determine the best model for the longitudinal relationship of each variable involved. These include: The baseline model (M1), which has an autoregressive path from the T1 to the T3 time point. The one-way prediction model (M2). That is, on the basis of the baseline model, it adds the path from activation to family function, recurrence risk perception, and health behavior decision; the path from family function to recurrence risk perception and health behavior decision; and the path from recurrence risk perception to health behavior decision. The one-way prediction model (M3). Namely, on the basis of the baseline model, it adds the path from family function to activation; the path from recurrence risk perception to activation and family function; and the path from health behavior decision to activation, family function, and recurrence risk perception. The integrated model (M4), which includes all paths from M1 to M3. Table 4 shows the fitting indexes of the four models and the results of model comparison.

**Table 4:** Model fitting and comparison results.

Models	χ^2^	df	CFI	TLI	RMSEA	∆χ^2^ (df) vs. M1	∆χ^2^ (df) vs. M4
M1	2374.248	42	0.932	0.914	0.096	–	–
M2	1302.156	30	0.972	0.967	0.044	1070.066 (12)^1)^	568.760 (12)^1)^
M3	1366.282	30	0.966	0.950	0.045	1008.948 (12)^1)^	630.872 (12)^1)^
M4	734.400	18	0.988	0.958	0.024	–	–

M1 is the baseline model, M2 and M3 are one-way prediction models, and M4 is the integrated model; ^1)^ means *P* < 0.01.

According to the model comparison results in Table 4, the baseline model (M1) was excluded because of insufficient fit. The comparative analysis indicated that the one-way prediction models M2 and M3 demonstrated significant improvement compared with M1 (∆CFI > 0.01), yet there was no evidence of equivalence between them. Compared with M2/M3, the integrated model M4 further optimized the model fit index (∆CFI > 0.01). Based on the model comparison results, M4 achieved the best fit while maintaining the theoretical construct relationship and was determined as the final analysis model.

#### Path analysis of cross-lag model

**Figure 1:**
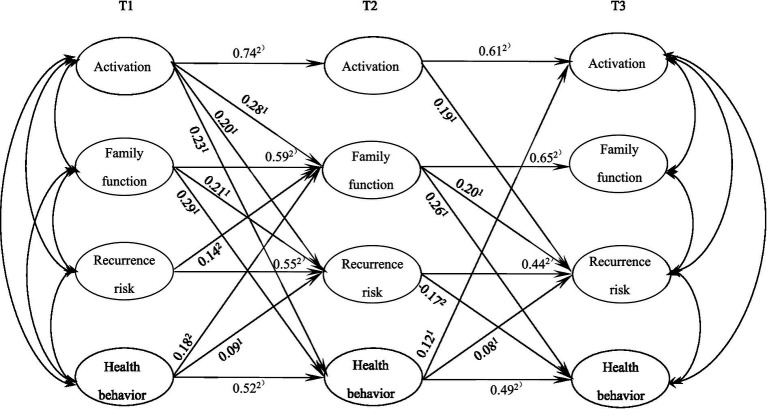
Cross-lag model. Only significant standardized paths are presented, ^1)^*P* < 0.01, ^2)^*P* < 0.001.

Figure 1 presents the cross-lagged model of activation, family functioning, relapse risk perception, and health behavior decision-making. In the cross-lag model, the autoregressive path of each variable at T1 – T3 was significant, and the variables at T1 and T2 could positively predict the variables at T2 and T3 (*p* < 0.01). T1 activation could significantly predict T2 family function (*β* = 0.28, *p* < 0.01), T2 recurrence risk perception (*β* = 0.20, *p* < 0.01), and T2 health behavior decision (*β* = 0.23, *p* < 0.01). T1 family function could significantly predict T2 recurrence risk perception (*β* = 0.21, *p* < 0.01) and T2 health behavior decision-making (*β* = 0.29, *p* < 0.01). T2 activation significantly positively predicted T3 recurrence risk perception (*β* = 0.19, *p* < 0.01). T2 family function could significantly positively predict T3 recurrence risk perception (*β* = 0.20, *p* < 0.01) and could significantly positively predict T3 health behavior decision (*β* = 0.26, *p* < 0.01). Recurrence risk perception at T2 significantly positively predicted T3 health behavior decision (*β* = 0.17, *p* < 0.01), and health behavior decision at T2 significantly positively predicted T3 activation (*β* = 0.12, *p* < 0.01). Bootstrap analysis showed that T2 family function (*β* = 0.073, *p* < 0.01) and T2 recurrence risk perception (*β* = 0.034, *p* < 0.01) had significant indirect effects on T1 activation in the prediction of T3 health behavior decision. These results indicated that family function and recurrence risk perception had a longitudinal mediating effect on the mechanism of activation on health behavior decision-making.

### Sensitivity analysis

Building upon the original cross-lagged model, I re-ran the analysis after including age, gender, stroke type, comorbidities, and education level as control variables.

The results indicate that, after controlling for the aforementioned covariates, the significance and direction of all significant cross-lagged paths in the original model remained essentially unchanged. In particular, the key coefficients forming the mediation pathway—such as the prediction of T1 activation on T2 family function and perceived risk of recurrence, and the prediction of T2 family function and perceived risk of recurrence on T3 health behavior decision-making—remained statistically significant (*p* < 0.05).

Robustness of the Mediation Effects: Bootstrap analysis based on the new model revealed that the longitudinal mediation effects of family function (*β* = 0.068, 95% CI [0.032, 0.110]) and perceived risk of recurrence (*β* = 0.029, 95% CI [0.010, 0.055]) remained significant (confidence intervals did not include zero). This demonstrates that, even after controlling for potential confounders, the mediating mechanism—where activation promotes health behavior decision-making by enhancing family function and risk perception—remains robust.

## Discussion

### The health behavior decision and activation of stroke patients interact with each other

Buelow et al. showed that patients with high activation had a clearer perception of disease risks and rehabilitation benefits, which prompted them to actively follow medical advice, such as taking regular medication and adhering to rehabilitation training (29). At the same time, positive emotions reduce the risk of recurrence through physiological mechanisms such as reducing stress hormone levels and improving vascular endothelial function, and enhance self-efficacy, making it easier for patients to overcome rehabilitation obstacles (30). The social support system further enhances patients’ enthusiasm for participation through emotional support and behavioral demonstration. The continuous implementation of healthy behaviors decision-making strengthens activation by improving functional status and quality of life, forming a positive cycle (31). This interaction ultimately encourages patients to make more scientific health decisions, reduce the risk of recurrence, and improve long-term prognosis. T2 healthy behavior decision improves T3 activation intensity through behavior reinforcement feedback. Adhering to healthy behaviors decision-making, such as taking regular medication or undergoing rehabilitation training, can improve physiological function and enhance self-efficacy, thereby strengthening patients’ confidence (32). At the same time, the objective benefits obtained during behavior execution, such as symptom relief or functional recovery, further verify the correctness of the decision-making and promote a continuous increase in activation (33). This dynamic interaction enables patients to participate more actively in subsequent interventions, forming a virtuous cycle of “decision – behavior – feedback – reinforcement”. This suggests that clinical medical staff should focus on early positive feedback design to optimize healthy behaviors decision-making and improve patients’ long-term compliance through quantifiable benefits.

### Family function and relapse risk perception had a longitudinal mediating role in the influence mechanism of activation on health behavior decision-making

Utilizing a longitudinal tracking design, this study conducted an in-depth investigation into the dynamic evolutionary mechanisms among positivity, family functioning, relapse risk perception, and health behavior decision-making. Data analysis revealed significant cross-temporal predictive effects among the variables, presenting a complex causal chain characterized by multistage and multidimensional interactions.

First, regarding the longitudinal predictive effects, the study found that positivity at T1 exhibited extensive prospective predictive power. It not only directly predicted concurrent outcomes (at T2)—specifically family functioning, relapse risk perception, and health behavior decision-making—but its effects also permeated into the subsequent phase. Specifically, T1 positivity significantly facilitated the optimization of T2 family functioning and mitigated T2 relapse risk perception, thereby laying the groundwork for health behavior decisions at T2. Furthermore, variables at T2 demonstrated distinct self-perpetuating and transformative characteristics: T2 positivity predicted T3 relapse risk perception; T2 family functioning not only directly predicted T3 relapse risk perception but also determined the quality of T3 health behavior decisions; conversely, T2 health behavior decisions positively fed back to sustain and enhance T3 positivity. These findings suggest that early intervention strategies should prioritize enhancing patients’ positive psychological resources (positivity) and repairing their family support systems. By constructing a virtuous cycle of “positive mindset → family support → risk cognition → behavioral change,” interventions can fundamentally improve long-term prognostic trajectories (34, 35).

Second, in the detailed analysis of intrinsic mechanisms, Bootstrap mediation effect tests revealed more refined transmission pathways. The results indicated that family functioning (*β* = 0.073, *p* < 0.01) and relapse risk perception (*β* = 0.034, *p* < 0.01) at T2 played crucial chained mediating roles in the relationship between T1 positivity and T3 health behavior decision-making. This implies that positive psychological resources do not directly influence long-term behaviors; rather, they must pass through two consecutive stages—“empowering the family” and “adjusting cognition”—to maximize their utility. Specifically, T1 positivity enhances T2 family functioning (i.e., strengthening informational support and emotional bonds among members) (36), effectively buffering excessive worries about disease deterioration, thereby reducing T2 relapse risk perception, and ultimately optimizing T3 health behavior decisions. This process constructs a chained mediation path of “positive mindset → family support → risk cognitive adjustment → behavioral change,” fully demonstrating that positive mental states must be externalized into concrete social support networks and internalized into rational risk assessments to effectively drive persistent health behavior changes (37, 38).

Furthermore, this study substantiated the “dual-path” driving mechanism of family functioning. Beyond the indirect chained effects mentioned above, the direct effect of T2 family functioning on T3 health outcomes was equally significant. Data showed that T2 family functioning could not only directly predict T3 health behavior decision-making (*β* = 0.26, *p* < 0.01) but also indirectly influence behavioral decisions by enhancing patients’ vigilance toward relapse (i.e., positively predicting T3 relapse risk perception, *β* = 0.20, *p* < 0.01). This reveals that the impact of the family system on patients is characterized by the unity of knowledge and action: on one hand, good family functioning directly strengthens patients’ executive capacity for health behaviors by providing supervision and encouragement; on the other hand, it elevates patients’ awareness of relapse risk by fostering a health-focused family atmosphere. This “support-cognition-behavior” dual-path mediation mechanism jointly amplifies the cumulative impact of early positivity on long-term health decisions (39, 40). Theoretically, this also explains why, in clinical practice, mere psychological counseling often fails to translate into sustained behavioral change without the coordination of the family system.

In conclusion, future clinical interventions should extend beyond individual-level psychological counseling to synchronously strengthen the construction of family support systems and the scientific management of relapse risk perception. By adopting a “dual-path synergy” strategy, interventions can maximize the long-term adherence to health behaviors among patients.

### Limitations of the study

This study has several limitations that warrant attention. First, the sample was confined to a single regional population, which may increase sampling error; thus, future research should employ multicenter, large-sample designs to validate the generalizability of these conclusions. Second, the reliance on self-reported outcomes may have introduced reporting bias to some extent. Additionally, while this study revealed the chained mediating effect of family functioning and relapse risk perception, the model did not account for potential confounding from variables such as self-efficacy. Crucially, key clinical variables, including the severity of neurological deficits and depressive symptoms, were not controlled for in this study. These factors are likely to influence the relationships among the studied variables, and therefore, future investigations should incorporate them to ensure more robust and precise findings.

## Conclusion

Within 6 months after stroke, there is a reciprocal predictive relationship between activation and health behavior decision-making in stroke patients. Family function and perceived risk of recurrence have a longitudinal mediating effect on the mechanism by which activation influences health behavior decision-making. Consequently, clinical nursing practice urgently needs to move beyond traditional, singular psychological counseling models and implement more targeted, integrated intervention strategies. Specifically, healthcare professionals should strive to establish a virtuous cycle of “positive mindset → family support → risk cognition → behavioral change.” On one hand, by empowering the family system and strengthening informational support and behavioral supervision among members, the “dual-path” effect of family functioning can be leveraged to directly enhance behavioral execution and optimize risk perception. On the other hand, guiding patients to establish a rational perception of relapse risk can translate positive psychological resources into tangible health management motivation. This “dual-path synergy” strategy, which synchronously strengthens family support and risk cognition management, effectively addresses the challenge of translating psychological resources into sustained behavioral change. Thus, it provides a vital theoretical foundation and practical paradigm for developing precision nursing protocols aimed at reducing stroke recurrence rates and improving long-term health behavior adherence in patients.

## Data Availability

The original contributions presented in the study are included in the article/supplementary material, further inquiries can be directed to the corresponding author.
